# CB1 receptor blockade counters age‐induced insulin resistance and metabolic dysfunction

**DOI:** 10.1111/acel.12438

**Published:** 2016-01-13

**Authors:** Christopher Lipina, Lobke M. Vaanholt, Anastasija Davidova, Sharon E. Mitchell, Emma Storey‐Gordon, Catherine Hambly, Andrew J. Irving, John R. Speakman, Harinder S. Hundal

**Affiliations:** ^1^ Division of Cell Signalling and ImmunologySir James Black CentreSchool of Life SciencesUniversity of DundeeDundeeUK; ^2^ Institute of Biological and Environmental SciencesUniversity of AberdeenAberdeenUK; ^3^ Division of NeuroscienceMedical Research InstituteNinewells HospitalUniversity of DundeeDundeeUK; ^4^ Institute of Genetics and Developmental BiologyChinese Academy of Sciences, ChaoyangBeijingChina

**Keywords:** aging, cannabinoid receptor type 1, insulin resistance, rimonabant

## Abstract

The endocannabinoid system can modulate energy homeostasis by regulating feeding behaviour as well as peripheral energy storage and utilization. Importantly, many of its metabolic actions are mediated through the cannabinoid type 1 receptor (CB1R), whose hyperactivation is associated with obesity and impaired metabolic function. Herein, we explored the effects of administering rimonabant, a selective CB1R inverse agonist, upon key metabolic parameters in young (4 month old) and aged (17 month old) adult male C57BL/6 mice. Daily treatment with rimonabant for 14 days transiently reduced food intake in young and aged mice; however, the anorectic response was more profound in aged animals, coinciding with a substantive loss in body fat mass. Notably, reduced insulin sensitivity in aged skeletal muscle and liver concurred with increased CB1R mRNA abundance. Strikingly, rimonabant was shown to improve glucose tolerance and enhance skeletal muscle and liver insulin sensitivity in aged, but not young, adult mice. Moreover, rimonabant‐mediated insulin sensitization in aged adipose tissue coincided with amelioration of low‐grade inflammation and repressed lipogenic gene expression. Collectively, our findings indicate a key role for CB1R in aging‐related insulin resistance and metabolic dysfunction and highlight CB1R blockade as a potential strategy for combating metabolic disorders associated with aging.

## Introduction

Insulin plays a prominent role in the modulation of glucose homeostasis and energy metabolism. Consequently, impaired responsiveness to insulin, termed insulin resistance, is a key component of the metabolic syndrome and a prelude to type 2 diabetes mellitus (T2DM). Importantly, a number of studies have now linked aging with development of insulin resistance in rodents as well as obese and nonobese individuals (Rowe *et al*., 1983; O'Shaughnessy *et al*., 1992). However, those factors which contribute to aging‐related insulin resistance and metabolic dysfunction remain poorly understood.

Initiation of insulin‐induced signalling stems from the activated insulin receptor kinase which can bind and regulate a number of downstream intracellular targets, including the insulin receptor substrate (IRS) family of proteins and phosphatidylinositol 3 kinase (PI3K) (Johnston *et al*., 2003). The subsequent PI3K‐mediated generation of phosphatidylinositol‐(3,4,5)‐triphosphate (PIP3) promotes translocation of protein kinase B (PKB, also known as Akt) to the plasma membrane where it undergoes phosphorylation/activation by upstream kinases at its two key regulatory sites, Thr308 and Ser473 (of PKBα/Akt1), respectively. Activated PKB/Akt stimulates glycogen synthesis and GLUT‐dependent glucose uptake by phosphorylating and inactivating GSK3 (glycogen synthase kinase 3) and the Rab GTPase‐activating protein AS160 (Akt substrate of 160 kDa), respectively (Cross *et al*., 1995; Sano *et al*., 2003). Proper regulation of these cellular processes is therefore crucial for maintaining glucose homeostasis by controlling glucose storage and utilization.

Growing evidence indicates that the endocannabinoid system (ECS) is intimately involved in the physiological control of food intake and energy expenditure, through its ability to target central and peripheral sites including skeletal muscle, liver and adipose tissue (Pagotto *et al*., 2006). Many of the biological actions of the ECS are mediated through G‐protein‐coupled cannabinoid type 1 (CB1R) and type 2 (CB2R) receptors which can be activated by several endogenous ligands, including anandamide (AEA) and 2‐arachidonoylglycerol (2‐AG). Importantly, ECS dysregulation has been linked to abdominal obesity and other risk factors for type 2 diabetes (Engeli *et al*., 2005; Osei‐Hyiaman *et al*., 2005; Matias *et al*., 2006). For example, genetic and diet‐induced obese animal models display elevated endocannabinoid levels in the hypothalamus and peripheral tissues (Di Marzo *et al*., 2001; Osei‐Hyiaman *et al*., 2005; Matias *et al*., 2006). Furthermore, increased circulating levels of AEA and 2‐AG, as well as elevated levels of 2‐AG within visceral adipose tissue, have been reported in obese and/or hyperglycaemic type 2 diabetic patients (Bluher *et al*., 2006; Matias *et al*., 2006). In accord with this, hyperactivation of CB1R‐induced signalling has been implicated in numerous metabolic abnormalities including hyperphagia, insulin resistance, glucose intolerance and impaired lipid homeostasis (Engeli *et al*., 2005; Jbilo *et al*., 2005; Osei‐Hyiaman *et al*., 2005). Conversely, genetic or pharmacological CB1R blockade improves metabolic status, for example by promoting reductions in body weight and fat mass, as well as enhancing insulin sensitivity and glucose tolerance. Indeed, these beneficial responses may be mediated through central appetite suppression and/or through direct modulation of peripheral energy metabolism (Bensaid *et al*., 2003; Ravinet Trillou *et al*., 2003, 2004; Jbilo *et al*., 2005; Pagotto *et al*., 2006; Osei‐Hyiaman *et al*., 2008; Jourdan *et al*., 2010).

Despite advances in our understanding of metabolic regulation by the ECS, as well as previous reports of age‐related deregulation of cannabinoid receptors and endocannabinoid hydrolysing enzymes (Pascual *et al*., 2013, 2014), little is known regarding the involvement of this signalling system in aging‐associated metabolic dysfunction. Herein, we explored the effects of chronic (14 day) administration of rimonabant (SR141716), a potent and selective CB1R inverse agonist (Landsman *et al*., 1997), on the energy budgets of young (4 month old) and aged (17 month old) adult male mice. Physical activity (PA) and body temperature (Tb) were monitored throughout the 14‐day experiment using implanted transmitters, and resting metabolic rate (RMR) by indirect calorimetry. This protocol permitted investigation of the effects of chronic rimonabant treatment upon different components of energy intake and expenditure, as well as body weight and fat mass. In addition, the impact of rimonabant upon key metabolic signalling pathways was determined in skeletal muscle, adipose tissue and liver isolated from young and aged mice. We show that aging was associated with a marked reduction in skeletal muscle and liver insulin sensitivity, which coincides with increased body fat mass. Intriguingly, this aging‐related insulin desensitization concurred with increased muscle and hepatic CB1R gene expression, indicative of enhanced endocannabinoid/CB1R tone within these tissues. Strikingly, rimonabant improved insulin sensitivity and glucose tolerance in aged, but not young, adult mice. Moreover, CB1 blockade induced a marked loss in body fat mass, concomitant with its ability to reduce adipogenic capacity and counteract low‐grade inflammation in aged epididymal fat tissue. Together, our findings reveal a novel role for the ECS in aging‐related metabolic dysfunction and identify CB1R as a potential therapeutic target in the treatment of metabolic disorders associated with the aging process.

## Results

### The anorectic and weight reducing effects of rimonabant are more profound in aged versus young adult C57BL/6 mice

**Figure 1:**
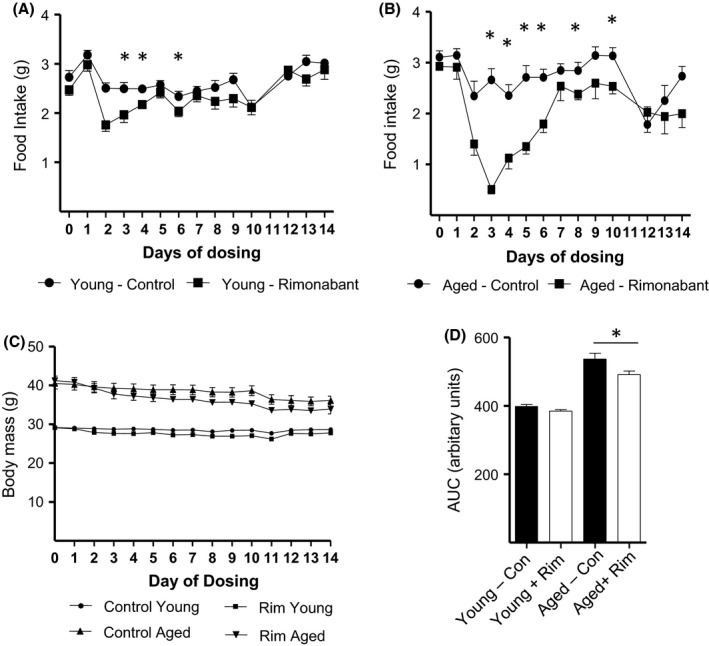
Changes in Food Intake and Body Weight of Male C57BL/6 Mice in Response to Rimonabant Administration. Mean daily food intake (A, B) and body mass (C, D) of young (A, C) and aged (B, D) C57BL/6 mice treated with rimonabant (Rim) or vehicle control (Con) in the absence and presence of insulin were monitored over a 14‐day period as shown. All values presented are the mean ± SEM from 15 mice. Asterisks denote statistically significant differences between vehicle control and SR141716 treated. *P < 0.05, ANOVA.

Rimonabant treatment significantly reduced daily food consumption in young mice from day 2 (2.5 ± 0.11 g to 1.8 ± 0.13 g; *P *< 0.05) until day 4 (2.5 ± 0.08 g to 2.1 ± 0.08 g; *P *< 0.05) of the treatment period (Fig. 1A). No significant differences in food intake were observed in response to the CB1R blocker in young mice for the remainder of the 14‐day study period. Strikingly, rimonabant triggered a more profound and prolonged decrease in daily food consumption in aged mice (Fig. 1B), including a marked reduction from 2.7 ± 0.22 g to 0.51 ± 0.09 g (*P *< 0.05) at treatment day 3. This preceded a more protracted recovery period, whereby daily food intake was significantly reduced until day 10, excluding treatment days 7 and 9. It should be noted that the observed reduction in food consumption in young and aged control‐treated mice on days 10 and 11 was likely attributable to the fasting period which preceded the glucose tolerance test on day 11. In accord with its more robust anorectic response in aged mice, rimonabant also promoted a greater reduction in total body mass in aged animals compared to younger counterparts (Fig. 1C). Corresponding statistical analysis of the AUC (area under the curve) revealed a significant 8.5% decrease in the AUC representing aged animals treated with rimonabant versus control, compared to the 3.5% (not significant) reduction in AUC mediated by CB1R blockade in young mice (Fig. 1D). It should be noted that there were no significant differences between vehicle control and rimonabant treatment groups at baseline in both young and aged mice.

### Rimonabant does not alter parameters of energy expenditure in young and aged mice

**Figure 2:**
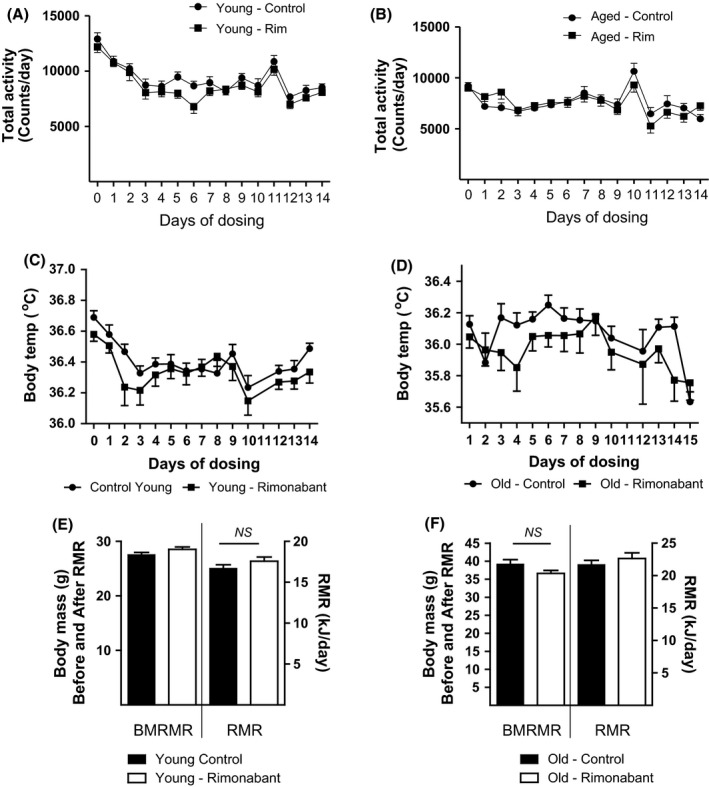
Effects of Rimonabant administration on Key Indicators of Energy Expenditure in Young and Aged Mice. Young and aged adult male C57BL/6 mice treated with rimonabant (Rim) or vehicle control (Con) had their physical activity (A, B) and body temperature (C, D) monitored daily during the 14‐day study period as indicated. Mean resting metabolic rates (RMR) determined on day 9 of the study are also shown (E, F). All values presented are the mean ± SEM from 15 individual animals. *NS*, not significant, ANCOVA.

Next, we explored the possibility that the observed reductions in body mass in response to rimonabant may also be associated with increased systemic energy expenditure (Zhang *et al*., 2012). For example, rimonabant has been previously reported to increase energy expenditure in diet‐induced obese C57BL/6 mice (Zhang *et al*., 2012). However as shown in Fig. 2, neither physical activity (Fig. 2A and B), body temperature (Fig. 2C and D) or resting metabolic rate (RMR) (Fig. 2E and F), were significantly altered by rimonabant treatment in young and aged mice. The hyperactivity on days 10 or 11 coincided with removing the food prior to performing the GTT. Therefore, these data indicate that it is unlikely that CB1R blockade promotes reductions in body weight through altering energy expenditure in young or aged mice.

### Aging‐related insulin resistance in skeletal muscle and liver coincides with increased CB1R gene expression

**Figure 3:**
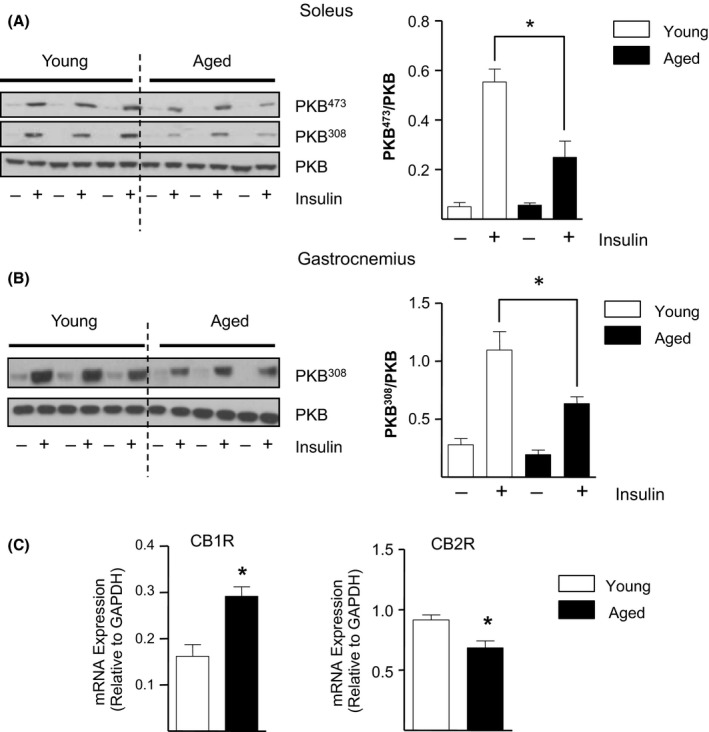
Aging‐related Insulin Resistance in Skeletal Muscle is Associated with Reduced Insulin Sensitivity and Upregulated CB1R Gene Expression. Lysates prepared from Soleus (A) and Gastrocnemius muscle (B) from young and aged mice stimulated with or without insulin were immunoblotted using phospho and native PKB/Akt antibodies as indicated. *n *=* *5 per group, *P < 0.05, *t*‐test. Alternatively, total RNA extracted from noninsulin‐treated gastrocnemius muscle was used to determine CB1R and CB2R mRNA abundance by qPCR analysis (C). Relative mRNA values presented are the mean ± SEM from 5 individual animals. *P < 0.05, *t*‐test.

Numerous studies have linked aging with development of insulin resistance (Rowe *et al*., 1983; O'Shaughnessy *et al*., 1992; Sczelecki *et al*., 2013). In accord with this, we report a marked reduction in the ability of insulin to phosphorylate/activate PKB at its two key regulatory sites Thr^308^ (by 1.7‐fold; *P *< 0.05) and Ser^473^ (by 1.6 fold; *P *< 0.05) in aged versus young soleus tissue (Fig. 3A). In addition, aged gastrocnemius muscle also displayed a significant 1.7‐fold decrease in insulin‐induced PKB/Akt^473^ phosphorylation relative to tissue from younger mice (Fig. 3B). Strikingly, this aging‐related impairment in insulin sensitivity coincided with a 1.8‐fold (*P *< 0.05) increase in CB1R mRNA abundance in aged versus young gastrocnemius muscle, in contrast to the 1.3‐fold (*P *< 0.05) decrease in CB2R mRNA (Fig. 3C). In addition, analysis of respective liver samples similarly revealed a significant 1.7‐fold reduction in insulin‐induced PKB^473^ phosphorylation in aged versus young tissue (Fig. S1A), concurring with a marked 3.8‐fold (*P *< 0.05) increase in hepatic CB1R gene expression (Fig. S1B). Therefore, these data indicate that impaired insulin action in aged skeletal muscle and liver is associated with enhanced ECS/CB1R tone.

### Rimonabant improves insulin sensitivity and glucose tolerance in aged but not young mice

**Figure 4:**
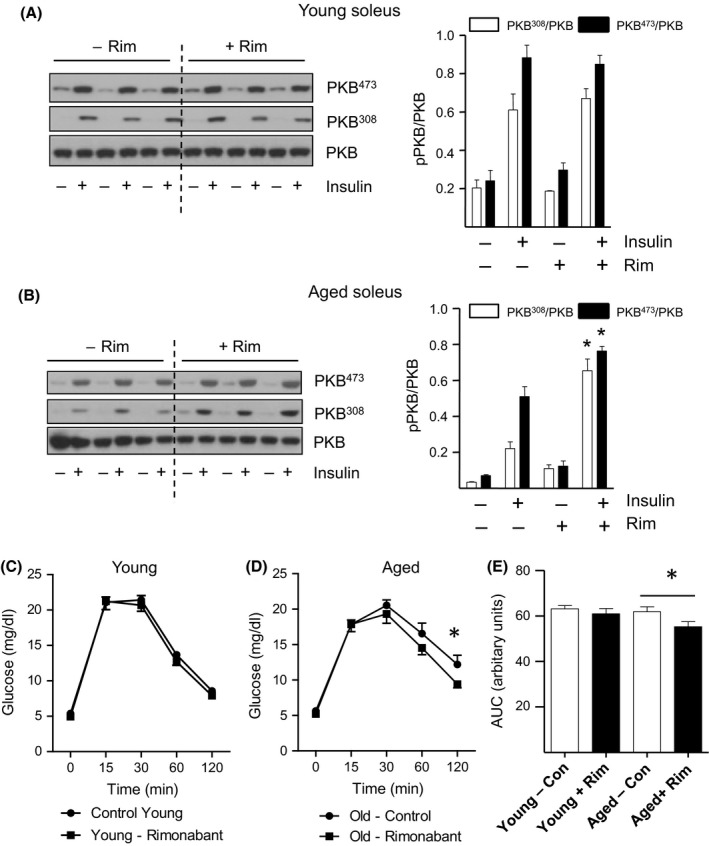
Rimonabant Improves Muscle Insulin Sensitivity and Glucose Tolerance of Aged But Not Young Mice. Lysates prepared from solei of young (A) and aged (B) mice stimulated with or without insulin following treatment with rimonabant (Rim) or vehicle control (Con) were immunoblotted using phospho and native PKB/Akt antibodies as indicated. Values presented are the mean ± SEM from 5 individual animals. Asterisks denote a statistically significant difference versus corresponding insulin‐stimulated control. *NS*, not significant. *P < 0.05, *t*‐test. The GTT was performed in young (C) and aged (D) mice administered with rimonabant (Rim) or vehicle control (Con) as described in the Methods. *P < 0.05, ANOVA. Corresponding area under the curve (AUC) data were calculated and presented using arbitrary units (E). Values presented are the mean ± SEM from 15 individual animals. *P < 0.05, ANOVA.

Based on our observation that reduced insulin sensitivity in skeletal muscle and liver concurs with enhanced ECS/CB1R tone, we hypothesized that CB1R antagonism may act to ameliorate aging‐related insulin desensitization. Strikingly, rimonabant treatment significantly enhanced insulin‐stimulated PKB/Akt^308^ and PKB/Akt^473^ phosphorylation by 3‐fold and 1.5‐fold respectively, in aged soleus muscle (Fig. 4B), but not in solei of younger mice (Fig. 4A). Moreover, treatment with the CB1R blocker also augmented insulin‐induced PKB/Akt^473^ phosphorylation (by 1.8‐fold; *P *< 0.05) in aged gastrocnemius muscle (Fig. S2).

Allied to its insulin‐sensitizing action within aged skeletal muscle, rimonabant also significantly enhanced insulin‐stimulated PKB/Akt^473^ phosphorylation (by 1.8 fold) in aged livers, in contrast to conveying a slight repressive (although not significant) effect in hepatic tissue from younger mice (compare Fig. S3A and B). Importantly, in accord with these improvements in insulin‐induced signalling in aged skeletal muscle and liver, we further report enhanced glucose tolerance in aged mice administered rimonabant, as determined by GTT (Fig. 4D). Intriguingly, this improvement in glucose tolerance was not manifested in younger mice treated with the inverse CB1R agonist (Fig. 4C). Statistical analysis revealed an 11% decrease in the AUC representing aged animals treated with rimonabant versus control (*P *< 0.05), compared to the 3.4% (not significant) reduction in response to CB1R blockade in younger mice (Fig. 4E). Moreover, this rimonabant‐mediated improvement in glucose tolerance coincided with the partial (~50%) normalization of fasting plasma insulin levels, which were found to be 3.8‐fold (*P *< 0.05) higher in aged versus young mice (Fig. S5A). Together, these findings indicate enhanced insulin sensitivity following rimonabant administration in aged mice, and reveal age‐dependent effects of CB1R inhibition upon insulin action and systemic glucose homeostasis.

### Rimonabant downregulates abundance of the catalytic subunit of PP2A in skeletal muscle

**Figure 5:**
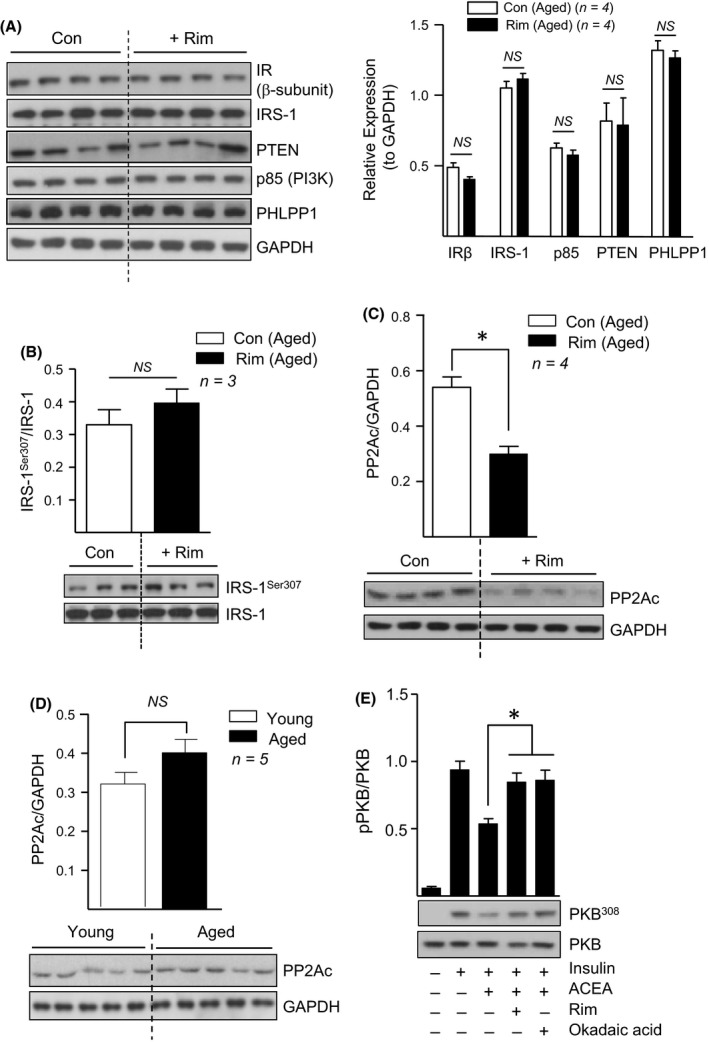
Rimonabant‐mediated Improvement in Insulin Sensitivity Coincides with Repressed PP2A in Skeletal Muscle. Gastrocnemius muscle tissue lysates from young and aged mice treated with rimonabant (Rim), or vehicle control (Con) as indicated, were immunoblotted using the antibodies shown (A–D). In addition, L6 myotubes were incubated with 1 μm ACEA, 100 nm rimonabant and/or 100 nm okadaic acid in serum‐free medium prior to stimulation with insulin or vehicle control (20 nm for 10 min) (E). Resulting cell lysates were immunoblotted using phospho (Thr 308) and native PKB/Akt antibodies. Values presented are the mean ± SEM from 3 independent experiments. *, *P < *0.05, *t*‐test. *NS,* not significant.

In an attempt to elucidate the mechanism(s) by which rimonabant acts to improve insulin sensitivity, we first explored the possibility that CB1R blockade may function by positively modulating upstream insulin receptor signalling. However, as shown in Fig. 5A, administration of rimonabant did not alter protein abundance of the insulin receptor β subunit, IRS‐1, PTEN, or the p85 regulatory subunit of PI3K in aged gastrocnemius muscle. Moreover, CB1R inhibition did not significantly alter phosphorylation of IRS‐1 at Ser307 (Fig. 5B), a site whose phosphorylation in response to CB1R activation has been associated with suppressed IRS‐1/PKB dependent signalling (Liu *et al*., 2012). Indeed, this is consistent with our previous work demonstrating the ability of rimonabant to enhance insulin sensitivity in rat L6 myotubes, an *in vitro* skeletal muscle model, without altering upstream IRS‐1/PI3K signalling (Lipina *et al*., 2010). Therefore, we next focussed on alternative pathways involving the potential modulation of PHLPP1 and PP2A (protein phosphatase 2A), two key negative regulators which act to dephosphorylate and inactivate PKB/Akt (Resjo *et al*., 2002; Liu *et al*., 2012). Rimonabant treatment did not alter levels of PHLPP1 protein in aged gastrocnemius muscle (Fig. 5A) but did, however, promote a significant 1.8‐fold reduction in the protein abundance of the PP2A catalytic subunit (PP2Ac) in the same tissue (Fig. 5C). Notably, we also observed a modest (1.3‐fold) increase in PP2Ac protein abundance in gastrocnemius muscle of aged versus young mice, although this was not found to be significant (Fig. 5D). Allied to these observations *in vivo*, we further demonstrate that the ability of the selective CB1R agonist ACEA to inhibit insulin‐induced PKB/Akt^308^ phosphorylation in L6 myotubes is completely prevented by co‐treatment with either rimonabant or okadaic acid, a potent PP2A inhibitor (Fig. 5E). Together, these data implicate PP2A as a key component in CB1R‐induced insulin resistance, whereby its repression in response to CB1R blockade may serve to alleviate its inhibitory action towards PKB/Akt.

### Rimonabant conveys anti‐adipogenic and anti‐inflammatory effects in aged epididymal fat tissue

**Figure 6:**
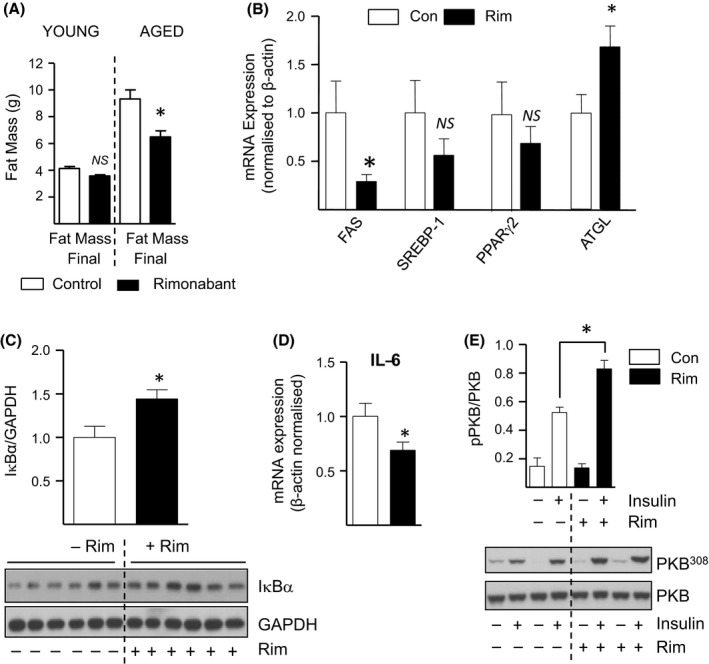
Rimonabant Reduces Body Fat Mass and Conveys both Anti‐Adipogenic and Anti‐Inflammatory Responses in Adipose Tissue of Aged Mice. (A) Final body masses of rimonabant (Rim)‐ and vehicle control (Con)‐treated mice were determined by DXA analysis. *n *=* *15 for each group *P <* *0.05, ANOVA. *NS*, not significant. (B, D) The relative mRNA abundance of FAS (fatty acid synthase), SREBP‐1 (sterol regulatory element binding protein‐1), PPARγ2 (peroxisome proliferator‐activated receptor γ2), ATGL (adipose triglyceride lipase) and IL‐6 (interleukin‐6) were determined from total RNA extracted from epididymal fat tissue of aged mice treated with or without rimonabant as indicated. *n *=* *5 per group, *P <* *0.05, *t*‐test. (C, E) Lysates prepared from aged epididymal fat tissue of rimonabant‐ or vehicle control‐treated mice stimulated with or without insulin were immunoblotted using the antibodies indicated. All quantified values are presented as the mean ± SEM White unfilled bars represent vehicle control treated; black filled bars are rimonabant treated. Asterisks denote a statistically significant difference versus corresponding vehicle control treated. *P < 0.05, *t*‐test.

It is now widely acknowledged that obesity increases the risk of developing insulin resistance (Everhart *et al*., 1992). In accord with this, our DXA analysis revealed that body fat mass of aged mice was 2.3‐fold (*P *< 0.05) higher in comparison to their younger counterparts (Fig. 6A). Intriguingly, rimonabant treatment in aged mice led to a 1.5‐fold (*P *< 0.05) reduction in body fat mass, but exhibited no significant effect in younger animals (Fig. 6A). Consistent with its observed anti‐obesity action in older mice, rimonabant markedly decreased mRNA levels of the lipogenic gene fatty acid synthase (FAS) by 4‐fold (*P *< 0.05) in aged epididymal fat tissue (Fig. 6B). However, there was a tendency for rimonabant to reduce mRNA abundance of SREBP‐1 (sterol regulatory element binding protein ‐1), a transcriptional regulator of FAS, as well as the pro‐adipogenic gene PPARγ2 (peroxisome proliferator‐activated receptor γ), although the effects were not significant in each case (Fig. 6B). In addition, rimonabant treatment significantly induced mRNA abundance of the lipolytic enzyme ATGL (adipose triglyceride lipase) by 1.9‐fold (*P *< 0.05) (Fig. 6B). Therefore, these data indicate that CB1R blockade may act to reduce body fat in aged mice, at least in part, by impairing and/or inducing pro‐adipogenic and lipolytic processes, respectively.

Because adipose tissue is a key site for production and secretion of insulin‐desensitizing pro‐inflammatory cytokines, we subsequently explored the possibility that rimonabant may convey anti‐inflammatory actions within this tissue (Wang *et al*., 2011). As shown in Fig. 6C, we report a 1.5‐fold (*P *< 0.05) increase in the protein abundance of IκBα (Inhibitory Subunit of NF Kappa B alpha), a key repressor of NF‐κB‐driven pro‐inflammatory cytokine gene expression, within epididymal fat tissue of aged mice treated with rimonabant (Fig. 6C). Accordingly, this coincided with a significant 1.4‐fold reduction in the mRNA abundance of IL‐6 (Interleukin‐6), an established NF‐κB target gene (Fig. 6D), as well as a 1.3‐fold (*P *< 0.05) decrease in plasma IL‐6 levels (Fig. S5B). Moreover, in accord with its anti‐inflammatory response, rimonabant treatment was found to almost completely suppress protein abundance of the macrophage marker CD68 in aged epididymal fat tissue, indicative of reduced macrophage infiltration (Fig. S4). Notably, rimonabant has also been reported to increase expression of adiponectin, an insulin‐sensitizing adipokine, in adipose tissue of obese fa/fa rats and in cultured 3T3 F442A adipocytes (Bensaid *et al*., 2003). In accord with this, we demonstrate a significant 1.2‐fold (*P *< 0.05) increase in plasma levels of adiponectin following rimonabant treatment in aged mice (Fig. S5C). Therefore, the ability of CB1R antagonism to counteract low‐grade inflammation and/or increase levels of adiponectin may, at least in part, contribute to its insulin‐sensitizing action in aged epididymal fat, as demonstrated by the 1.5‐fold (*P *< 0.05) augmentation in insulin‐induced PKB^308^ phosphorylation (Fig. 6E). Importantly, these findings indicate anti‐adipogenic, pro‐lipolytic and anti‐inflammatory actions conveyed by rimonabant within aged adipose tissue which may, at least in part, underlie its ability to improve systemic insulin sensitivity and glucose tolerance in older mice.

## Discussion

A number of animal studies and clinical trials have previously described the anti‐obesity and insulin‐sensitizing effects of CB1R antagonism (Ravinet Trillou *et al*., 2003; Jbilo *et al*., 2005; Alonso *et al*., 2011). This study reveals for the first time, a protective role for CB1R inhibition against aging‐related metabolic dysfunction in mice. We report that rimonabant improves insulin sensitivity and glucose tolerance in aged mice, whereas the CB1R blocker failed to convey these same beneficial responses in younger animals. This coincided with the ability of rimonabant to induce a more profound anorectic response and greater reduction in body fat mass in older mice. Crucially, we demonstrate increased CB1R gene expression in aged skeletal muscle and liver, indicative of enhanced endocannabinoid/CB1R tone in response to aging. Notably, our findings are consistent with previous work reporting high fat diet‐induced increases in CB1R tissue expression in younger animals, together with the amelioration of dyslipidaemia and lipogenesis, as well as improved insulin sensitivity in mice deficient for CB1R (Osei‐Hyiaman *et al*., 2008). Indeed, it is possible that the improvements in insulin sensitivity that are conveyed by rimonabant in aged mice may be due, at least in part, to reductions in adiposity. However, it should be noted that previous studies utilizing hyperinsulinaemic‐euglycaemic clamps and/or ITT (insulin tolerance test), have documented the ability of rimonabant to improve insulin sensitivity without promoting significant reductions in body fat mass or visceral fat in db/db mice and diet‐induced obese dogs, respectively (Kim *et al*., 2012; Nam *et al*., 2012). Together with our previous work reporting direct insulin‐sensitizing actions by rimonabant in rat skeletal L6 myotubes (Lipina *et al*., 2010), these findings indicate that CB1R inhibition may at least partly enhance insulin sensitivity in aged mice independently of its ability to normalize age‐related adiposity.

Notably, rimonabant‐mediated insulin sensitization in aged skeletal muscle coincides with the ability of the CB1R blocker to repress expression of the catalytic subunit of PP2A, a key negative regulator of PKB/Akt (Andjelkovic *et al*., 1996). Moreover, pharmacological inhibition of PP2A, similar to rimonabant provision, were both shown to prevent CB1R‐induced insulin desensitization in rat skeletal L6 myotubes. In contrast, rimonabant treatment in aged mice failed to alter the expression of several key upstream insulin signalling components. In addition, we have previously reported the ability of rimonabant to enhance insulin sensitivity in L6 myotubes without impacting upon IRS‐1/PI3K signalling (Lipina *et al*., 2010). Therefore, these findings indicate that it is unlikely that CB1R blockade conveys its insulin‐sensitizing effects through modulating upstream signalling components, but instead, acts to alleviate PP2A‐mediated repression of PKB/Akt, at least in skeletal muscle.

In accord with its insulin‐sensitizing actions, the ability of rimonabant to reduce body fat was markedly enhanced in aged mice. This may be due, at least in part, to its more effective inhibition of food intake in aged animals, although the exact processes underlying this anorectic response remain unclear. One possible explanation may involve aging‐related increases in endocannabinoid/CB1R tone in regions of the brain associated with appetite regulation, such as the nucleus accumbens (Maldonado‐Irizarry *et al*., 1995; Winters *et al*., 2012). Indeed, these changes may be brought about by increased CB1R expression, or in response to the altered activity of enzymes implicated in the synthesis and/or degradation of endocannabinoids (Pascual *et al*., 2013). Consequently, such a scenario may convey the apparent augmentation of rimonabant's anorectic response in older mice. Furthermore, CB1R inhibition may counteract the appetite inducing effects of peptides such as ghrelin, whose circulating levels have been shown to increase with aging in mice (Senin *et al*., 2013; Lin *et al*., 2014).

CB1R blockade has also been shown to promote reductions in body fat mass through direct peripheral actions in adipocytes (Jbilo *et al*., 2005; Jourdan *et al*., 2010). In accord with this, we report the ability of rimonabant to repress or induce lipogenic and lipolytic genes, respectively, in aged epididymal fat tissue. Indeed, CB1R inhibition may act to diminish aging‐associated lipogenesis, a process known to be transduced in response to CB1R activation, as well as promoting increased lipolytic drive either directly or indirectly, for example through activation of the sympathoadrenal system (Jbilo *et al*., 2005; Molhoj *et al*., 2010). Furthermore, the anti‐inflammatory response conveyed by the CB1R blocker in aged epididymal fat may also contribute to its insulin‐sensitizing actions, in particular considering the fact that adipose tissue serves as a key site for the production and secretion of insulin‐desensitizing inflammatory cytokines such as IL‐6 and TNFα (Gnacinska *et al*., 2009; Gustafson, 2010).

Notably, previous pair‐feeding studies carried out in rodents have revealed that the beneficial metabolic effects conveyed by chronic (>1 week) pharmacological blockade of CB1R, or CB1R deficiency, may arise through food intake dependent or independent (e.g. increased energy expenditure) mechanisms (Cota *et al*., 2003; Jbilo *et al*., 2005; Herling *et al*., 2008; Nogueiras *et al*., 2008). Whilst this present study did not permit us to discriminate between these two different modes of action directly, our findings indicate that rimonabant is unlikely to convey its metabolic improvements in aged mice by increasing energy expenditure. Indeed, this was demonstrated by the lack of significant change in several parameters of energy expenditure including physical activity and resting metabolic rate following administration of the inverse CB1R agonist. However, we cannot rule out the possibility that rimonabant may act to enhance mitochondrial biogenesis and/or oxidative capacity within individual tissues, thereby contributing to their improved metabolic status (Jbilo *et al*., 2005; Tedesco *et al*., 2008). Indeed, to support this possibility we report a 2.5‐fold (*P *< 0.05) increase in PGC‐1α protein abundance in gastrocnemius muscle of aged mice treated with rimonabant, although no such increase was observed in aged epididymal fat tissue in response to CB1R inhibition (Fig. S6), thereby suggesting tissue‐specific actions. In an attempt to further address this issue, future work comparing the metabolic effects of rimonabant and peripherally acting CB1R antagonists (e.g. AM6545), which display low blood brain barrier penetration, may help delineate the relative contribution of hypophagic and peripherally targeted responses conveyed by CB1R blockade (Tam *et al*., 2010). Indeed, such studies may involve analysis of insulin signalling as well as utilizing hyperinsulinaemic‐euglycaemic clamps to assess insulin sensitivity *in vivo*. In addition, our findings also raise the question as to whether rimonabant promotes its beneficial responses by functioning to inhibit constitutive/tonic CB1R activity (i.e. inverse CB1R agonism), or alternatively, by acting as a receptor antagonist (Bouaboula *et al*., 1997; Xie *et al*., 2007). Follow up studies assessing the effects of CB1R stimulation and/or inhibition upon metabolic processes in primary cells (i.e. hepatocytes, muscle cells and adipocytes) isolated from young and aged animals, may prove useful in identifying age and/or tissue‐specific effects of CB1R antagonism/inverse agonism.

In conclusion, our findings reveal age‐dependent effects of CB1R inhibition upon metabolic function *in vivo*. We demonstrate that CB1R blockade leads to a marked reduction in food intake and body fat mass, concomitant with improved insulin sensitivity and glucose tolerance, in aged mice. Notably, these beneficial responses are either absent or less prominent in younger animals. Moreover, we report that insulin resistance in aged skeletal muscle and liver coincides with increased endocannabinoid/CB1R tone. Therefore, these findings reveal a previously unknown role for CB1R in conferring aging‐induced metabolic dysfunction and identify CB1R inhibition as a potential strategy to counteract age‐associated impairments in energy homeostasis.

## Experimental procedures

### Animal studies

Male C57BL/6 mice at 6 weeks of age (Charles River, MA, USA) were housed in a controlled 12 h light/dark environment and maintained *ad libitum* on a standard control diet (D12450B; Research Diets, New Brunswick, NJ, USA) until 4 months (young) or 16 months (aged) of age. At 16 months, mice were implanted intraperitoneally with temperature transmitters (PDT‐4000 E‐Mitter; Mini Mitter, Bend, OR, USA) under general anaesthesia (mixture of isoflurane and oxygen). Mice were allowed 14 days to recover prior to experimental treatment. Over the next 14 days, animals were administered 3% (v/v) ethanol in dH_2_O (vehicle control, *n *= 15) or SR141716 (10 μg g^−1^, *n *= 15) by oral gavage. During this time mice had *ad libitum* access to food and water, with body mass and food intake measured daily. The average body weights (mean ± SEM) of young control and rimonabant‐treated mice at the start of experimentation were 29.18 ± 0.45 g and 29.09 ± 0.39 g, respectively. The average body weights of aged control and rimonabant‐treated mice at the start of experimentation were 41.15 ± 1.43 g and 41.23 ± 1.17 g, respectively.

### Body temperature and general activity

Mice in their home cages were placed onto transponder energizers (ER‐4000 Receiver; Mini Mitter) allowing noninvasive monitoring of body temperature and physical activity throughout the study period. The VitalView Data Acquisition System (Mini Mitter) was used to collect the data at 1 min intervals.

### Resting metabolic rate (RMR)

RMR was determined in animals after 8–9 days of treatment using an open‐flow respiratory system described previously (Krol *et al*., 2003). All measurements took place during the light phase between 0700 and 1630. Briefly, fresh air was pumped through a sealed Perspex chamber containing the mouse and placed within an incubator set at 30 °C, within the thermo‐neutral zone for C57BL/6 mice (Speakman & Keijer, 2012). Air was pumped through the chamber at a rate of 500–700 mL min^−1^ which was monitored using an Alexander Wright DM3A flow meter. Air leaving the chamber was dried using silica gel and passed through a gas analyser (Servomex Xentra). Gas concentrations were measured continuously, and values recorded every 30 s for 180 min. Oxygen consumption rates were determined using the lowest 20 consecutive values (10‐min interval) and CO_2_ production over the highest 20 consecutive values (10 min interval). Data (mL O_2_ min^−1^) were converted to energy equivalents (kJ day^−1^) using the Weir equation and the mean respiratory quotient (RQ) for each group (VO_2_*(1.106 + 3.941/RQ)*4.184*60*24)/1000). Body masses were determined before and after each run.

### Dual‐energy X‐ray absorptiometry (DXA) analysis

Fat mass (FM) and fat free mass (FFM) of mice were determined twice at day 0 and day 14 using DXA (GE Medical Systems Ultrasound and BMD, Bedford, UK). Animals were anesthetized using an isoflurane/oxygen mixture for the duration of the scan (~3 min). Data were corrected using a calibration formula generated by linear regression of fat content as determined by DXA as previously described (Johnston *et al*., 2005).

### Glucose tolerance test

Following gavage on day 10, animals received a clean cage and food was removed from the hopper. The next morning a blood sample was taken *via* the tail tip to measure basal fasting glucose concentration (UltraTouch glucometer, LifeScan, UK). Mice were then injected intraperitoneally with glucose (2 g kg^−1^ body weight) and glucose concentration determined 15, 30, 60 and 120 min after injection. Mice were refed after the final measurement.

### Analysis of blood metabolites (plasma insulin, IL‐6 and adiponectin)

Fasting plasma insulin levels were measured using a Mercodia Insulin ELISA kit (Mercodia, Sweden) according to the manufacturer's instructions. Analysis of plasma IL‐6 and adiponectin was determined using Mouse IL‐6 and Adiponectin Quantikine^®^ ELISA Kits (both from R&D Systems, Abingdon, UK), respectively, according to the manufacturer's instructions.

### Dissection

On day 15, mice were culled by CO_2_ overdose. Half the mice received an injection of insulin (2 mU g^−1^) 10 min prior to cull. Careful excision of muscle (soleus and gastrocnemius), gonadal fat and liver was performed prior to being snap‐frozen in liquid nitrogen for storage at −80 °C. Blood serum specimens were also isolated and snap‐frozen.

### Muscle culture, cell/tissue treatments and analysis

Methods for culturing and treating rat L6 myotubes and their preparation for immunoblotting, RNA extraction, conventional RT–PCR and real‐time quantitative (qPCR) analysis have been previously described (Lipina *et al*., 2010; Turban *et al*., 2012). For animal tissue, frozen tissue was ground using a pestle and mortar prior to homogenization with ice‐cold lysis buffer. Resulting cell/tissue debris was removed from crude lysate by centrifugation at 3000 g for 10 min at 4 °C, and the resulting supernatant used for Western blot analysis. Proteins from cell/tissue lysates (30 μg) were subjected to SDS–polyacrylamide gel electrophoresis and immunoblotted as previously described (Lipina *et al*., 2010; Turban *et al*., 2012) with antibodies against IRS‐1 (Santa Cruz, CA, USA), phospho‐IRS‐1^Ser307^ (Merck‐Millipore), PHLPP1 (Merck‐Millipore, Darmstadt, Germany), PTEN (Santa Cruz), CD68 (Santa Cruz), p85‐PI3K (Merck‐Millipore), phospho‐PKB/Akt (Ser^473^ and Thr^308^; New England Biolabs, Hitchin, Herts, UK), native PKB/Akt (New England Biolabs), PGC‐1α (Abcam, Cambridge, UK), GAPDH (Sigma, Poole, Dorset, UK), insulin receptor β‐subunit (Merck‐Millipore) or the catalytic subunit of PP2A (PP2Ac, DSST, University of Dundee). Primary antibody detection was carried out using anti‐rabbit IgG‐HRP or anti‐mouse IgG‐HRP‐linked antibody (New England Biolabs) as appropriate by ECL. Resulting band intensities were quantified using ImageJ software (NIH, Bethseda, MD, USA).

### Statistical analysis

Data were analysed using GraphPad Prism (GraphPad, San Diego, CA, USA). Statistical analysis was performed using one‐way or two‐way ANOVA (with Bonferroni post hoc test) or Student's unpaired *t*‐test as appropriate and considered statistically significant at *P *<* *0.05. Metabolic rate data were analysed using ANCOVA as recommended by Tschop *et al*. (Tschop *et al*., 2012) using body composition parameters as covariates.

## Funding

This work was supported by the BBSRC and Diabetes UK.

## Conflict of interest

None declared.

## Author contributions

C.L. researched data/wrote and edited manuscript; L.V. researched data; A.D. researched data; E.G.‐S. researched data; C.H. researched data; A.J.I. contributed to discussion and edited manuscript; J.R.S. contributed to discussion and edited manuscript; H.S.H. wrote and edited manuscript.

## Supporting information


**Data S1** Experimental Methods.
**Table S1.** Primer Sequences used for qPCR analysis.
**Fig. S1** Aging–related insulin resistance in liver is associated with reduced insulin sensitivity and upregulated CB1R gene expression.
**Fig. S2** Rimonabant enhances insulin‐stimulated PKB/Akt Ser473 phosphorylation in aged gastrocnemius muscle.
**Fig. S3** Rimonabant enhances insulin sensitivity in liver of aged but not young mice.
**Fig. S4** Rimonabant reduces protein abundance of the macrophage marker CD68 in aged epididymal fat tissue.
**Fig. S5** Rimonabant‐induced changes in blood plasma insulin, interleukin‐6 and adiponectin levels in young and/or aged mice.
**Fig. S6** Rimonabant increases PGC‐1α Protein abundance in aged gastrocnemius muscle but not aged epididymal fat tissue.
