# CB1 receptor blockade counters age‐induced insulin resistance and metabolic dysfunction

**DOI:** 10.1111/acel.13378

**Published:** 2021-05-20

Christopher Lipina, Lobke M. Vaanholt, Anastasija Davidova, Sharon E. Mitchell, Emma Storey‐Gordon, Catherine Hambly, Andrew J. Irving, John R. Speakman and Harinder S. Hundal, *Aging Cell* (2016) **15**, 325–335. https://doi.org/10.1111/acel.12438, First published: 13 January 2016

In Lipina et al. (2016), the immunoblot data presented in Figure 3B (showing levels of phosphorylated PKB/Akt Thr308 and total PKB/Akt in gastrocnemius muscle of young and aged mice) have been duplicated in error from their previous original research article entitled, “NEU3 sialidase as a marker of insulin sensitivity: Regulation by fatty acids” published in the journal *Cellular* *Signalling* (see Figure 9A; Lipina C. et al. 2015. *Cellular* *Signalling* 27(9): 1742–1750). For clarification, both figures in question were generated using a common primary data set with additional data (*n* = 5 per experimental group) being included for the quantification of phospho/total PKB/Akt blot data shown in the bar graph in Figure 3B, in comparison with the *n* = 4 per experimental group indicated for the corresponding bar graph presented in Figure 9A of the research article published in *Cellular* *Signalling*.

The authors greatly regret this oversight in duplicating immunoblot data and provide clarification of the data presented in these two research articles in which, importantly, both show reduced insulin responsiveness in gastrocnemius muscle of aged versus young mice.
