# Supplementary material for: CB1 receptor blockade counters age‐induced insulin resistance and metabolic dysfunction
Source: Aging Cell. 2016 Jan 13;15(2):325–35. doi: 10.1111/acel.12438 (PMC4783351; doi:10.1111/acel.12438)
Supplement: Supplementary file 1 — Data S1 Experimental Methods. Table S1. Primer Sequences used for qPCR analysis. Fig. S1 Aging–related insulin resistance in liver is associated with reduced insulin sensitivity and upregulated CB1R gene expression. Fig. S2 Rimonabant enhances insulin‐stimulated PKB/Akt Ser473 phosphorylation in aged gastrocnemius muscle. Fig. S3 Rimonabant enhances insulin sensitivity in liver of aged but not young mice. Fig. S4 Rimonabant reduces protein abundance of the macrophage marker CD68 in aged epididymal fat tissue. Fig. S5 Rimonabant‐induced changes in blood plasma insulin, interleukin‐6 and adiponectin levels in young and/or aged mice. Fig. S6 Rimonabant increases PGC‐1α Protein abundance in aged gastrocnemius muscle but not aged epididymal fat tissue. [file ACEL-15-325-s001.pdf]

Supporting Information

Data S1: Experimental Methods

Total RNA Isolation and quantitative real-time PCR (qPCR) Analysis Total RNA was isolated from native tissues using Tri-Reagent® (Sigma Poole, UK) according to manufacturer's instructions. RNA was solubilized in nuclease-free H_2O and quantitated by measuring absorbance at 260 nm. For each sample, mRNA from 1 µg of total RNA was reverse transcribed using oligo(dT)18 primer and Moloney murine leukemia virus (M-MLV) reverse transcriptase (Promega, Madison, WI, USA). Quantitative PCR was performed using an ABI Prism 7000 (Applied Biosystems, Foster City, CA) real-time thermocycler and SYBR Green Quantitative RT-PCR kit (Sigma-Aldrich). PCR conditions were as follows: initial denaturation 95°C for 2 min followed by 40 cycles of denaturation at 95°C for 15 s, annealing at 55°C for 15 s, and extension at 68°C for 30 s. Relative fold changes in mRNA abundance versus GAPDH mRNA were calculated using a method described previously (Pfaffl 2001). The sequences of the primers used are included in Table 1.

Gene	Sense (5'-3')	Antisense (5'-3')
**CB1R**	CTGATGTTCTGGATCGGAGTC	TCTGAGGTGTGAATGATGATGC
**CB2R**	CCCTACCTGTAATCCCAGCA	TTGAGGTAAGGGGGTCTCAA
**IL-6**	CAAAGCCAGAGTCCTTCAGAG	GTCCTTAGCCACTCCTTCTG
**FAS**	GGCTGCAGTGAATGAATTTG	TTCGTACCTCCTTGGCAAAC
**SREBP-1**	ATGGACGAGCTGGCCTTCGGTGA GGCGGC	CAGGAAGGCTTCCAGAGAGGA
**PPARγ**	ATCTTAACTGCCGGATCCAC	AGGCACTTCTGAAACCGACA
**ATGL**	CTTCACGGGGTTCTCTGAGT	TCAACGGCTGAGCAACTCTA
**GAPDH**	TGGAAAGCTGTGGCGTGAT	GCTTCACCACCTTCTTGAT

Table 1. Primer Sequences used for qPCR analysis. CB1R; cannabinoid receptor type 1, CB2R; cannabinoid receptor type 2, IL-6; interleukin-6, FAS; fatty acid synthase, SREBP-1; sterol regulatory binding protein-1, PPARγ; peroxisome proliferator-activated receptor gamma, ATGL; adipose triglyceride lipase, GAPDH; glyceraldehyde 3-phosphate dehydrogenase

Supplementary Figure Legends

Supplementary Figure S1. Aging–related Insulin Resistance in Liver is Associated with Reduced Insulin Sensitivity and Upregulated Hepatic CB1R Gene Expression

(**A**) Liver tissue lysates from young and aged mice stimulated with or without insulin were immunoblotted using phospho (Ser 473) and native PKB/Akt antibodies as indicated. *n* = 5 per group, **P < 0.05*, *t*-test. (**B**) Alternatively, CB1R mRNA abundance from total RNA extracted from non-insulin treated liver tissue was determined by qPCR analysis. Relative mRNA values presented are the mean $\pm$ S.E.M. from 5 individual animals. Asterisks denote a statistically significant difference versus young as indicated. **P < 0.05*, *t*-test.

Supplementary Figure S2. Rimonabant Enhances Insulin-Stimulated PKB/Akt Ser473 Phosphorylation in Aged Gastrocnemius Muscle

Lysates prepared from gastrocnemius muscle of aged mice stimulated with or without insulin following treatment with rimonabant (Rim) or vehicle control (Con) were immunoblotted using phospho (Ser 473) and native PKB/Akt antibodies as indicated. Values presented are the mean $\pm$ S.E.M. from 4 individual animals. Asterisk denotes a statistically significant difference. **P* < 0.05, *t*-test.

Supplementary Figure S3. Rimonabant Enhances Insulin Sensitivity in Liver of Aged But Not Young Mice

Lysates prepared from livers young (**A**) and aged (**B**) mice stimulated with or without insulin following treatment with rimonabant (Rim) or vehicle control (Con), were immunoblotted using phospho (Ser473) and native PKB/Akt antibodies as indicated. Values presented are the mean $\pm$ S.E.M. from 5 individual animals. Asterisks denote a statistically significant difference versus corresponding insulin-stimulated control. *NS*, not significant. **P < 0.05*, *t*-test.

Supplementary Figure S4. Rimonabant Reduces Protein Abundance of the Macrophage Marker CD68 in Aged Epididymal Fat Tissue

Lysates prepared from aged epididymal fat tissue of rimonabant or vehicle control treated mice stimulated with or without insulin were immunoblotted using the anti-CD68 and anti-GAPDH antibodies as indicated.

Supplementary Figure S5. Rimonabant-Induced Changes in Blood Plasma Insulin, Interleukin-6 and Adiponectin Levels in Young and/or Aged Mice

Fasting plasma insulin (**A**), IL-6 (**B**) and adiponectin levels (**C**) were measured in young (**A**) and/or aged (**A-C**) mice administered with rimonabant (Rim) or vehicle control (Con) as indicated, and as described in the Methods. All values presented are the mean $\pm$ S.E.M. from 5 individual animals. *$P<0.05$, ANOVA.

Supplementary Figure S6. Rimonabant Increases PGC-1α Protein Abundance in Aged Gastrocnemius Muscle but Not Aged Epididymal Fat Tissue.

Lysates prepared from epididymal fat tissue (**A**) and gastrocnemius muscle (**B**) from aged mice treated with rimonabant (Rim) or vehicle control (Con), were immunoblotted using anti-PGC-1α and GAPDH antibodies antibodies as indicated. Values presented are the mean $\pm$ S.E.M. from 5 individual animals. Asterisks denote a statistically significant difference versus corresponding insulin-stimulated control. *NS*, not significant. *$P < 0.05$, *t*-test.

References for Supporting Information

Pfaffl MW (2001). A new mathematical model for relative quantification in real-time RT-PCR. *Nucleic Acids Res*. **29**, e45.

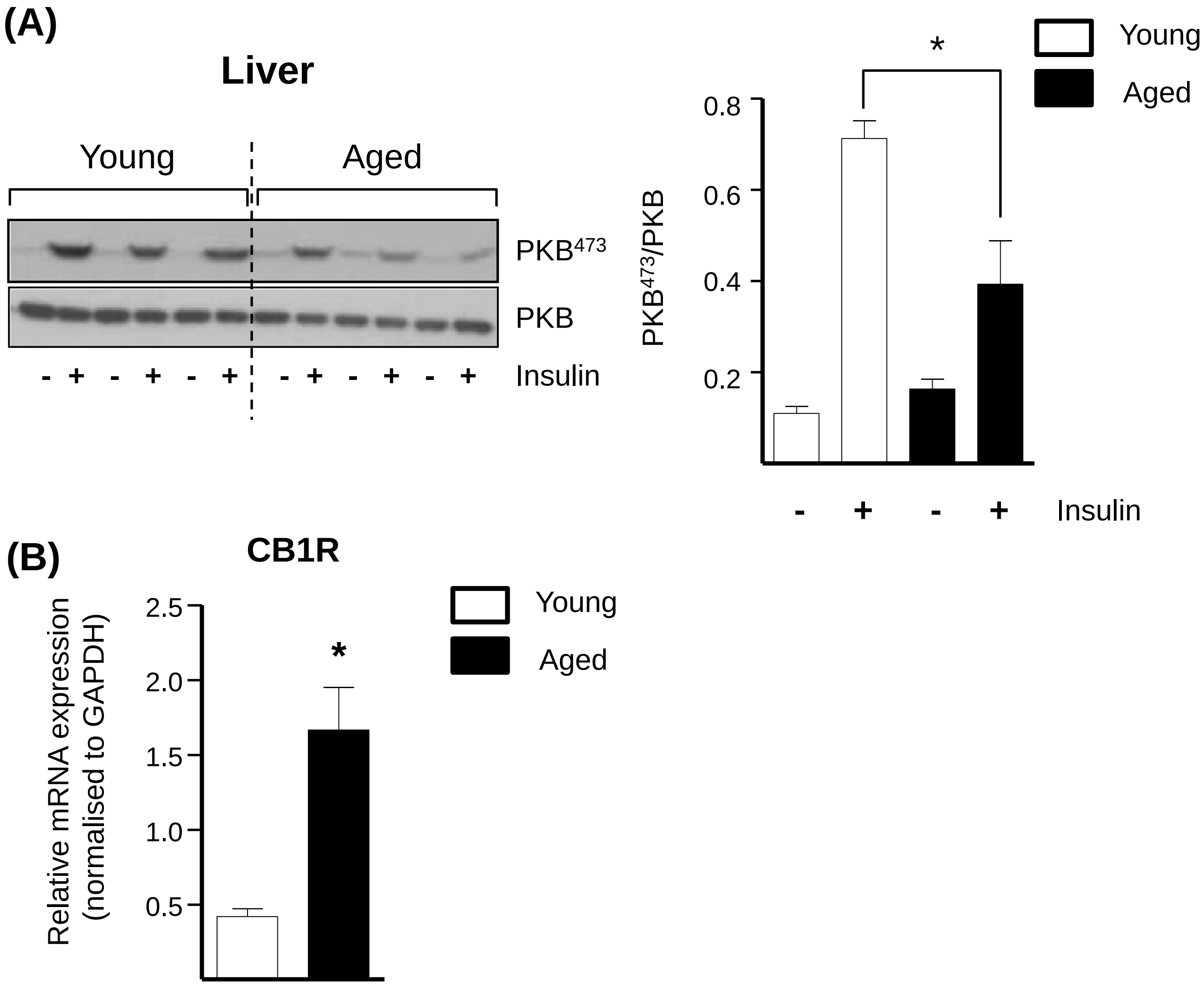

Supplementary Fig S1: Ageing–related insulin resistance in liver is associated with reduced insulin sensitivity and upregulated CB1R gene expression

(**A**) Liver tissue lysates from young and aged mice stimulated with or without insulin were immunoblotted using phospho (Ser 473) and native PKB/Akt antibodies as indicated. *n* = 5 per group, **P < 0.05*, *t*-test. (**B**) Alternatively, CB1R and CB2R mRNA abundance from total RNA extracted from non-insulin treated gastrocnemius muscle was determined by qPCR analysis. Relative mRNA values presented are the mean ± S.E.M. from 5 individual animals. Asterisks denote a statistically significant difference versus young (insulin treated for immunoblot quantifications). **P < 0.05*, *t*-test.

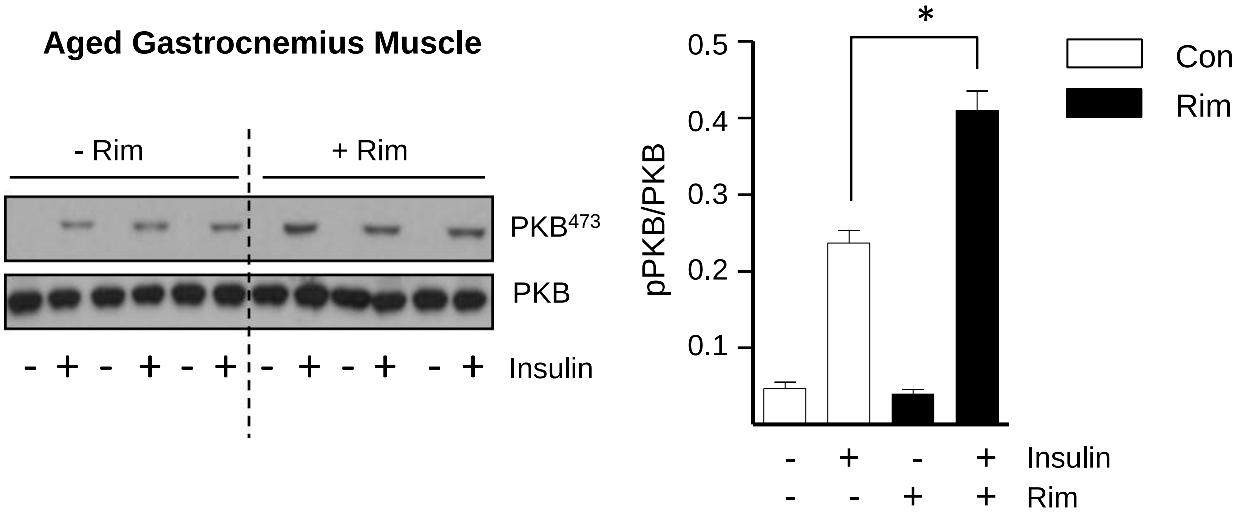

Supplementary Figure S2. Rimonabant Enhances Insulin-Stimulated PKB/Akt Ser473 Phosphorylation in Aged Gastrocnemius Muscle

Lysates prepared from gastrocnemius muscle of aged mice stimulated with or without insulin following treatment with rimonabant (Rim) or vehicle control (Con) were immunoblotted using phospho (Ser473) and native PKB/Akt antibodies as indicated. Bar graph data shows values presented as mean ± S.E.M. from 4 individual animals. Asterisk denotes a statistically significant difference. **P* < 0.05, *t*-test.

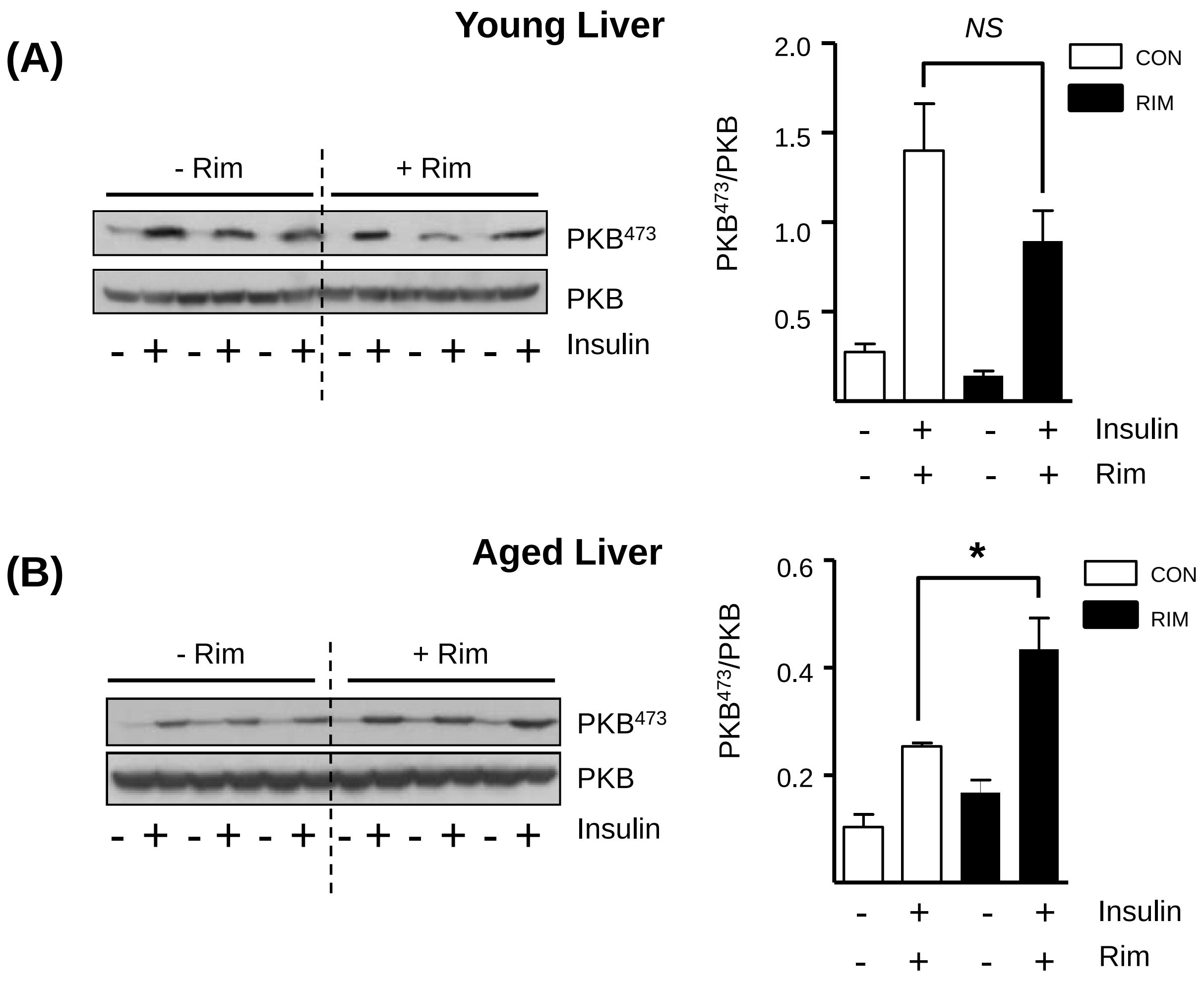

Supplementary Fig S3: Rimonabant enhances insulin sensitivity in liver of aged but not young mice

Lysates prepared from livers of young (**A**) and aged (**B**) mice stimulated with or without insulin following treatment with rimonabant (Rim) or vehicle control (Con), were immunoblotted using phospho and native PKB/Akt antibodies as indicated. Values presented are the mean $\pm$ S.E.M. from 5 individual animals. Asterisks denote a statistically significant difference versus corresponding insulin-stimulated control. *NS*, not significant. *$P < 0.05$, *t*-test.

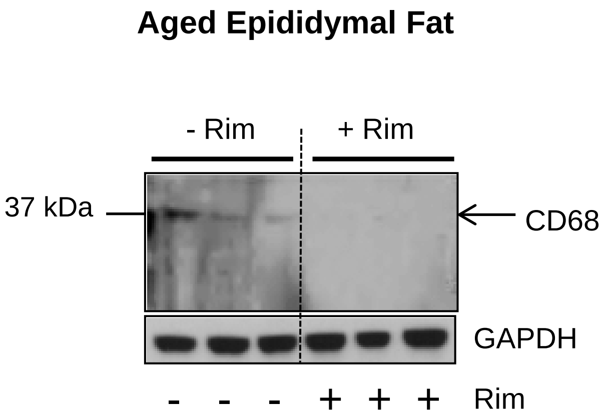

Supplementary Figure S4

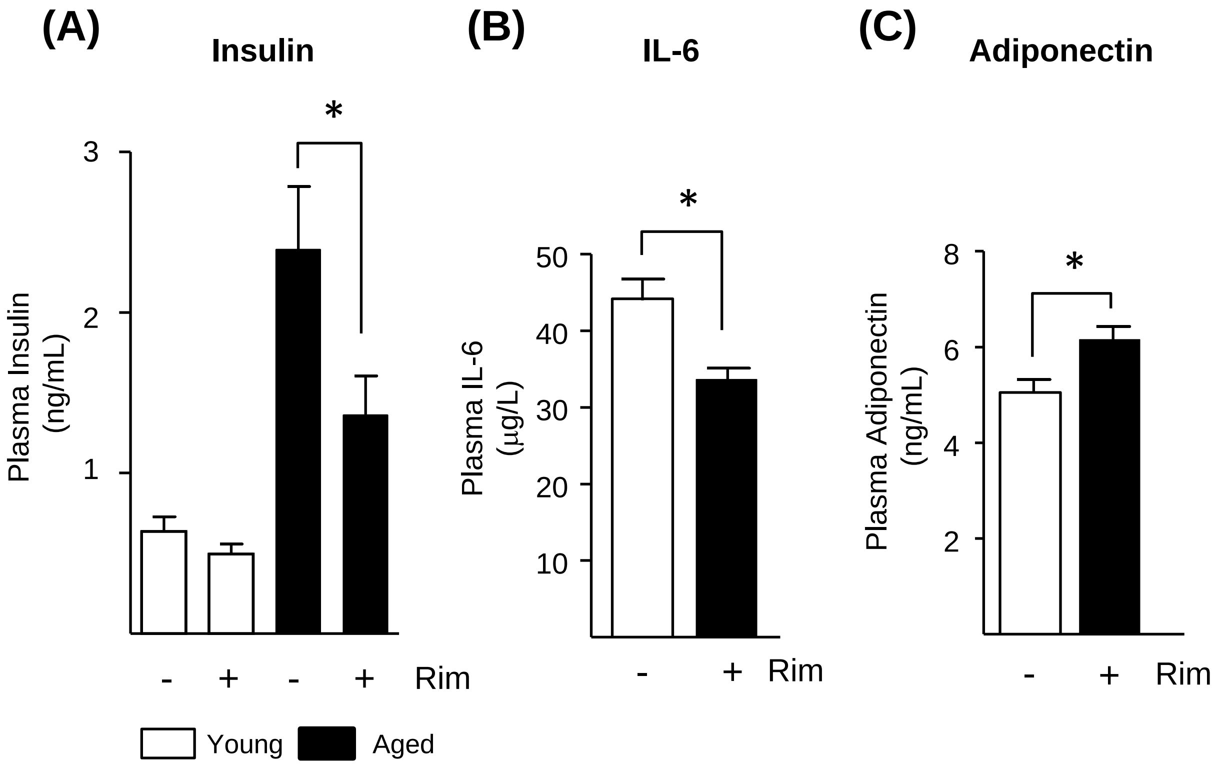

Supplementary Figure S5

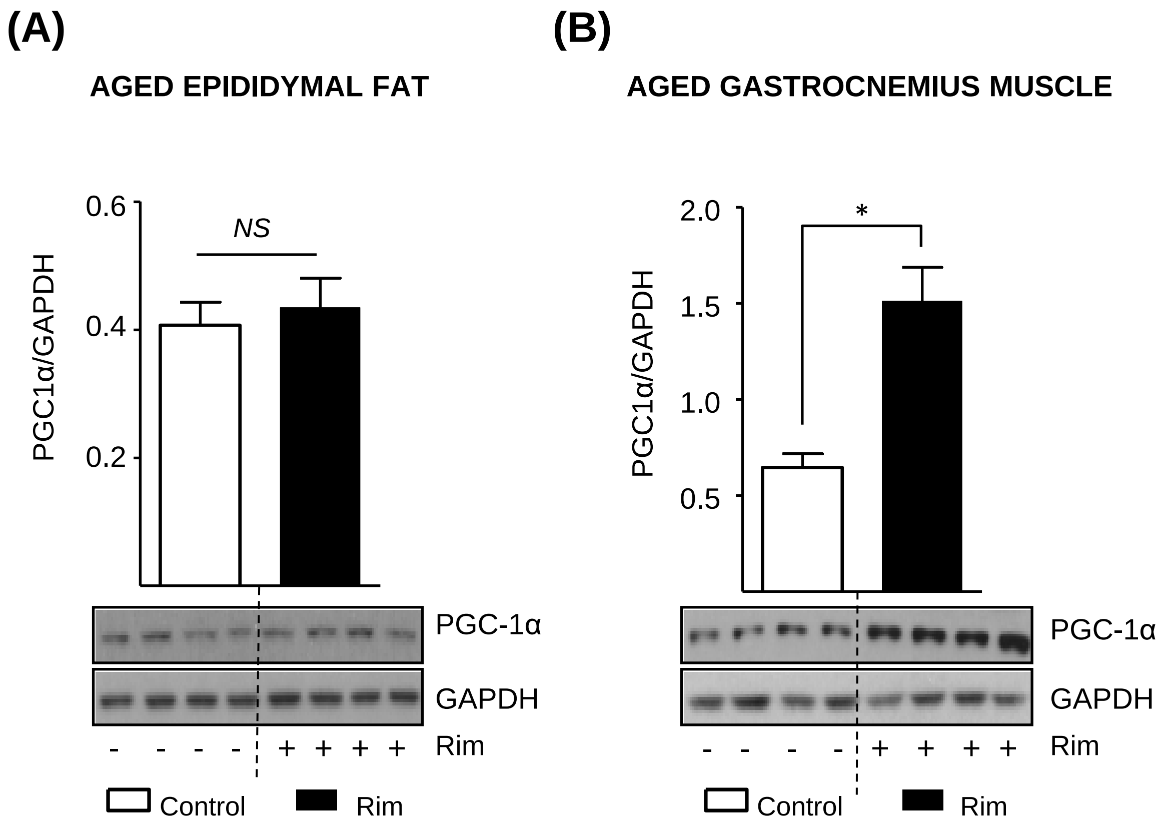

Supplementary Figure S6
